# Impacts of Tick Parasitism on the Rodent Gut Microbiome

**DOI:** 10.3390/microorganisms13040888

**Published:** 2025-04-12

**Authors:** Robert Jory Brinkerhoff, Joshua Pandian, Meghan Leber, Isabella D. Hauser, Holly D. Gaff

**Affiliations:** 1 Biology Department, University of Richmond, Richmond, VA 23173, USAbella.hauser@richmond.edu (I.D.H.); 2 Biology Department, Old Dominion University, Norfolk, VA 23529, USA; 3 School of Mathematics and Computer Science, University of KwaZulu-Natal, Durban 4041, South Africa

**Keywords:** Lyme disease, *Ixodes scapularis*, *Peromyscus leucopus*, *Sigmodon hispidus*, vector-borne disease, zoonoses

## Abstract

Host microbiota may impact disease vector behavior and pathogen transmission, but little is known about associations between ectoparasites and microbial communities in wildlife reservoir species. We used Illumina metagenomic sequencing to explore the impacts of tick parasitism on the rodent fecal microbiome in both a field and laboratory setting. We found that tick parasitism on wild hosts was associated with variation in the fecal microbiota of both the white-footed deermouse, *Peromyscus leucopus*, and the southern cotton rat, *Sigmodon hispidus*. In a lab experiment, we detected significant changes to the fecal microbiome after experimental exposure to immature ticks in treated versus control BALB/c mice. Whereas there is variation in the fecal microbiome associated with each of the host species we tested, some of the same microbial taxa, notably members of the family Muribaculaceae, occurred at higher relative abundance in tick-parasitized hosts in both the field and laboratory studies, suggesting that there are consistent impacts of tick parasitism on the host gut microbiome. We recommend future studies to test the hypothesis that epithelial cell secretions, generated as part of the host’s immune response to tick parasitism, could provide resources that allow particular microbial lineages in the mammalian gut to flourish.

## 1. Introduction

Links between gut microbial flora and health outcomes in general [1], as well as specific effects of microbial species composition on development [2], neurological disease [3], and behavioral responses to stress [4], are becoming increasingly apparent, as are impacts on endocrine signaling [5], gastrointestinal dysfunction [6,7], depression [8], and obesity [9]. In non-human hosts, infections such as parvovirus can result in dysbiosis which, in turn, can lead to additional negative health outcomes such as intestinal inflammation, impaired bile acid metabolism, and development of chronic enteropathies [10]. Studies relating gut microbiota and acute infectious disease are fewer in number than those that explore impacts of the microbiome on chronic conditions, but are yielding novel insights into disease processes [11]. Round and Mazmanian [12] reviewed studies of mouse models and humans, and concluded that elements of the microbiome play a key role in protecting the host from potential pathogens either through immune system modulation or production of metabolites that reduce the likelihood of infection. More recent studies have tied changes in human microbiome composition to development of sequalae from emerging diseases like SARS-CoV-2 infection [13] and Lyme disease [14].

Whereas recent studies have demonstrated interactions between the host microbiome and vector-borne infections [15,16,17], little is known about how vectors themselves may interact with the host’s microbiome. The host microbiome may affect the likelihood of encountering or being parasitized by a vector through a number of potential mechanisms. Host-associated bacteria have been hypothesized to mediate volatile organic compounds, which may serve as important attractants to parasites such as flies, mosquitoes, and ticks [18,19,20]. Altered gut microbiome composition has also been implicated in inflammatory skin diseases [21,22,23]. Atopic dermatitis may be linked to an increase in abundance in the gut of *Clostridium difficile* or decrease in Bifidobacterium [21,22], while bacteria from the groups Clostridiales and Erysipelotrichales have been linked with changes in immune function, resulting in psoriasis [21]. As skin physiology and chemistry can affect attractiveness to ectoparasites [24], factors that affect the gut microbiome may influence ectoparasite burden.

Alternatively, the gut microbiome may affect, and be affected by, feeding and foraging behavior. Perry et al. [9] demonstrated that dysbiosed mice show different feeding behaviors than mice with unaltered microbiomes and, given that foraging behavior in mammals may be linked to tick exposure [25], the gut microbiome, through mediation of physiological condition, could indirectly affect ectoparasite exposure. Andre et al. [26] explored variation in the liver microbiome of *Peromyscus leucopus*, the principal reservoir of the Lyme disease pathogen *Borrelia burgdorferi* [27], but did not report any links between microbial community composition and infection with B. burgdorferi or another genus of vector-borne zoonoses, *Bartonella*. Whether parasitism impacts the host’s gut microbiome is similarly understudied. Internal parasites such as cestodes have been shown to affect the vertebrate gut microbiome, as well as expression of host immune genes [28], and it has been hypothesized that eradication of helminth parasites from humans has resulted in an altered gut microbiome and potentially led to increased incidence of autoimmune disease [29]. Whether an ectoparasite, through immune-mediated or other mechanisms, can im-pact the host microbiome is currently unknown.

Our goal was to assess links between variation in the host gut microbiome and exposure to ticks in both field- and lab-based studies to explore host–parasite interactions that may impact transmission of vector-transmitted pathogens. Specifically, we tested the hypotheses that (1) parasitism by ticks would be related to variation in the gut microbiome of wild rodents, and (2) experimental exposure to ticks would result in consistent and predictable changes in the host’s gut microbiome. In the field, we sampled the white-footed deermouse, *Peromyscus leucopus*, and the cotton rat, *Sigmodon hispidus* (a potential reservoir for the agent of Tidewater spotted fever, *Rickettsia parkeri* [30,31]), and compared gut microbiome composition among parasitized and unparasitized individuals. In the lab, we conducted an artificial tick infestation experiment to collect microbiome data from lab mice before and after experimentally induced tick parasitism.

## 2. Materials and Methods

**Figure 1:**
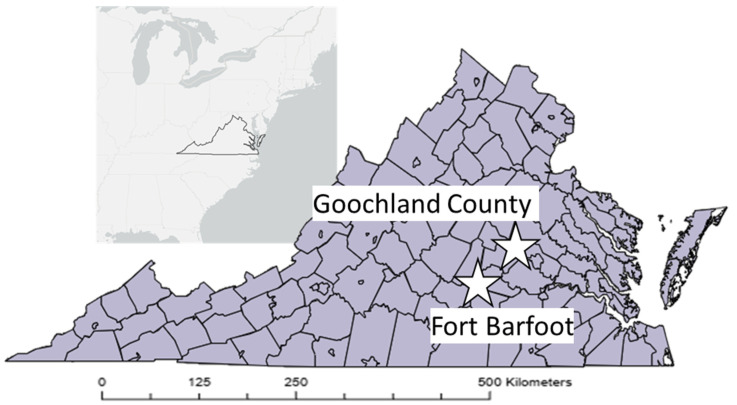
Locations of the field sites in central Virginia from which wild rodent fecal samples were collected.

We sampled wild rodents for ongoing research on zoonotic pathogen transmission at two sites in central Virginia: a University of Richmond-owned property in eastern Goochland County; and Ft. Barfoot, which straddles Dinwiddie, Brunswick and Nottaway Counties (Figure 1).

We sampled early successional open-canopy habitats at each site, where we set eight transects of 10 Sherman live traps with 10 m spacing between traps and 150–1500 m between transects. Traps were baited with bird seed, and set for three consecutive nights, once every three weeks for four trapping sessions per site. Traps were checked by 10:30 each morning and left open, but not set, between sampling sessions. Captured rodents were marked using individually numbered ear tags, and data on species, sex, and weight were recorded prior to release. While rodents were in hand, we collected all visible ticks from the head, neck, ears, limbs, and axillae using fine pointed forceps; individuals were examined for 30–60 s following collection of the last tick, or in the event that no ticks were detected. We collected fecal samples directly into sterile 1.5 mL flip-cap microcentrifuge tubes that were kept on dry ice during multi-day sampling sessions before being frozen dry at −20 °C upon return to the lab. All animal work for the field project was reviewed and approved by the Institutional Animal Care and Use Committee of the University of Richmond (protocol 20-01-001).

In fall 2022, we ordered 10 female Balb/c mice of at least 7 weeks of age from Envigo (now Inotiv, Inc., Lafayette, IN, USA) for use in a parasitism experiment. Mice were held in individual mouse cages in the animal facility at the University of Richmond for approximately one week prior to the initiation of tick parasitism. Mice were assigned randomly to one of two experimental treatment groups for the parasitism trials, with initial fecal samples collected as described above prior to the first tick application. Tick applications were performed as follows: mice were held by the scruff and approximately 50–70 larval *Ixodes scapularis*, sourced from the Oklahoma State University tick colony, were applied to ears, head, ventrum, and dorsum of all individuals in the treatment group with a fine-bristled paintbrush. Individuals in the control group underwent a mock exposure identical to the tick application, but with no ticks on the paintbrush. Animals were then returned to clean cages with a wire mesh metabolic floor instead of shredded pine bedding for seven days. Cages were checked twice per day for engorged ticks, which were collected into sterile microcentrifuge tubes, and cleaned once per day. After the seven-day engorgement period, mice were moved to cages with shredded pine bedding for approximately five days prior to a second round of tick exposure, which was performed exactly like the first, except that animals were sacrificed at the end of the second tick exposure with a second fecal sample collected from each animal prior to euthanasia. All laboratory animal work was reviewed and approved by the Institutional Animal Care and Use Committee of the University of Richmond (protocol 20-06-002).

### 2.1. Molecular Methods

We extracted DNA from all individual fecal samples using commercial DNA isolation kits (Macherey-Nagel Blood and Tissue Kit, Macherey-Nagel, Inc., Bethlehem, PA, USA) using manufacturer’s protocols. We used different metagenomic sequencing protocols for the field and lab samples. For the 2020 field samples, we amplified a portion of the V4 hypervariable region of the 16S rRNA gene using conserved universal primers 515F (modified from [32]) and 806R (modified from [33]), and followed the Illumina 16S metagenomics library preparation guidelines [34] using an Illumina Nextera XT Index kit (Illumina, Inc., San Diego, CA, USA) through the library pooling step. Pooled libraries from up to twenty-five samples were loaded into an Illumina iSeq 100 i1 Reagent v2 (300-cycle) cartridge and run on an Illumina iSeq 100 instrument according to the manufacturer’s instructions, and with 5 µL of 100 pM PhiX positive control library added to 15 µL of the pooled library. Due to changes in resource availability during the project, sequencing of a portion of the V3-V4 hypervariable 16S region for fecal DNA extracts from the lab experiment was conducted on an Illumina MiSeq (2 × 250 reads) following Nextera library preparation by Novogene, Inc. (Novogene Corp., Sacramento, CA, USA). Sequencing of a portion of the V3-V4 hypervariable 16S region for fecal DNA extracts from the lab experiment was conducted on an Illumina MiSeq (2 × 250 reads) following Nextera library preparation by Novogene, Inc. (California, USA).

### 2.2. Informatic and Statistical Analyses

Sequence data were processed using Qiime2 [35] as follows: raw reads were imported, demultiplexed, truncated to remove loci with low read quality, and denoised/quality filtered with DADA2 [36], implemented in Qiime2 [35]; raw reads were truncated (but not trimmed) to 150 bases for the wild rodents and, for the lab mouse data, we truncated sequences (but did not trim them) at 225 and 222 bases for forward and reverse reads, respectively. We used weighted taxonomic classifiers [37] based on the Silva 138 rRNA reference database [38], and trained on the animal distal gut [39] to identify amplicon sequence variants (ASVs) for both the V4 and V3-V4 datasets. We assessed alpha and beta diversity outputs (e.g., unifrac analysis [40], rarefaction, evenness and diversity estimates) from Qiime2 and compared microbial community composition among metadata features (e.g., rodent species, sampling site, and presence/absence of ticks for the field data and treatment group and timepoint for the lab experiment) by perMANOVA using the R package vegan [41] implemented in Qiime2 [35]. We assessed relative over- or underrepresentation of taxa based on metadata features using ANCOM-BC [42] implemented in Qiime2 [35]. For these analyses, we implemented a prevalence cut-off of 0.5% and a significance threshold of 0.1.

## 3. Results

We collected fecal samples from 45 individual wild rodents sampled at two field sites and representing two species; one species (*Sigmodon hispidus*) was only detected at the Fort Barfoot site, whereas the other species (*Peromyscus leucopus*) was detected at both sites (Table 1).

**Figure 2:**
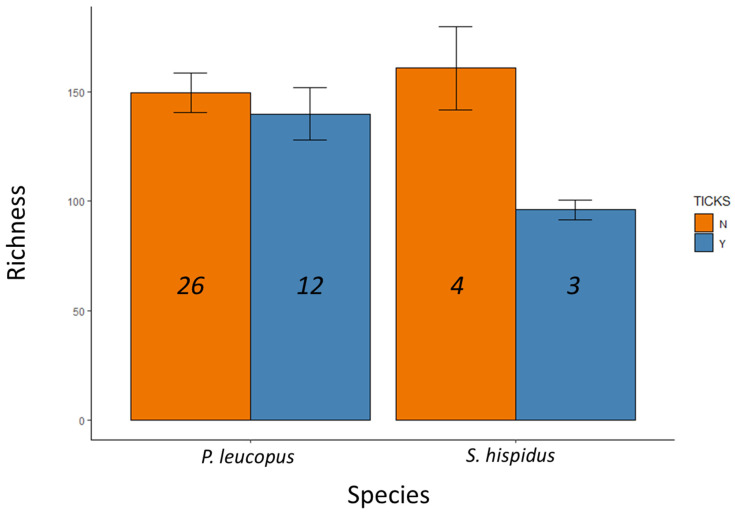
Microbial taxonomic richness detected in fecal samples from two wild rodent species and as a function of tick parasitism (Y indicates animals with ticks, N indicates animals without detected ticks). Numbers in each bar represent sample sizes and error bars represent standard error.

Ticks were present on 15 individuals (Table 1), but these tick samples were lost before they could be identified or counted. Some individuals were captured more than once, but only the fecal sample from first collection was used for library preparation and analysis. Metagenomic libraries from these 45 samples generated a median read depth of 96,488 and a range of 24,996–312,172 reads per sample (Table 1). We identified 12,530 amplicon sequence variants (ASVs) that could be classified into 666 species-level-or-higher taxa. Taxonomic richness did not vary by rodent species (*F* = 082, *p* = 0.37), but did vary depending on whether the host was parasitized by ticks (*F* = 4.21, *p* = 0.047), with no significant interaction between host species and tick parasitism (*F* = 2.31, *p* = 0.14; Figure 2).

**Figure 3:**
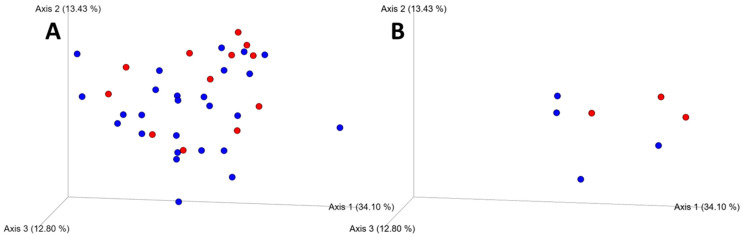
Emperor principal coordinates analysis (PCoA) plots of weighted unifrac distances showing variation in microbial community assemblages for tick-parasitized (red) as well as un parasitized (blue) *P. leucopus* (panel **A**) and *S. hispidus* (panel **B**).

Microbial community composition, as characterized by weighted unifrac [40] distances, varied significantly by rodent species (*F* = 4.15, *p* = 0.007), sampling location (*F* = 3.0, *p* = 0.002), and tick parasitism (*F* = 1.78, *p* = 0.049; Figure 3).

In addition to main effects, there were significant interactions between rodent species and sampling location (*F* = 3.07, *p* = 0.006), and sampling location and presence of ticks (*F* = 2.14, *p* = 0.017), although these interactions are confounded by the lack of *S. hispidus* samples from the Goochland, Virginia site.

**Figure 4:**
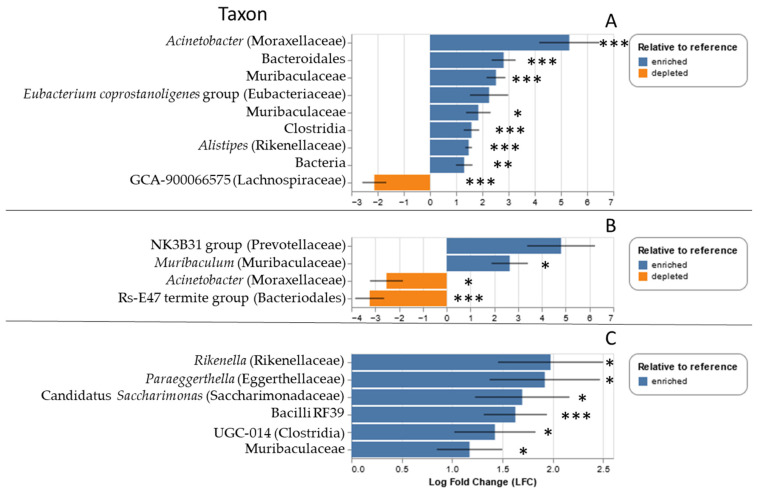
Relative abundance of particular microbial taxa over- (blue) or underrepresented (orange) in tick parasitized (**A**) *S. hispidus*, (**B**) *P. leucopus* at the Fort Barfoot site, and (**C**) *P. leucopus* at the Goochland site. Taxa are listed by genus, where possible, with family or next highest taxonomic group in parenthesis. * *q* < 0.05, ** *q* < 0.005, *** *q* < 0.0005.

Particular microbial taxa varied in relative abundance for both *P. leucopus* and *S. hispidus* depending on whether the individual was parasitized by ticks. For *S. hispidus*, members of the genera *Acinetobacter* and *Alistipes*, as well as multiple taxa within the Muribaculaceae, were overrepresented in tick parasitized hosts, whereas one member of the Lachnospiraceae (GCA-900066575) was underrepresented (all *q*-values < 0.01; Figure 4A). For *P. leucopus*, the following taxa were overrepresented at the Goochland site: *Rikenella*, *Paraeggerthelia*, candidatus *Saccharimonas*, Clostridia UGC-014, and a members of the Muribacculaceae and family RF39 (all *q*-values < 0.05, Figure 4B). At the Ft. Barfoot site, only one taxon was overrepresented in tick-parasitized *P. leucopus* (Muribaculaceae, *q* = 0.044), whereas two (*Acinetobacter* (*q* = 0.02) and a member of the family Rs-E47 termite group of the Bacteriodales (*q* << 0.001) were underrepresented (Figure 4C).

**Figure 5:**
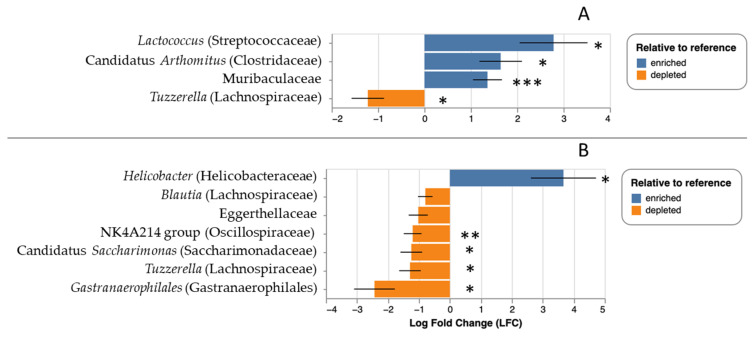
Relative abundance of particular microbial taxa over- (blue) or underrepresented (orange) in laboratory mice at the end of the experimental treatment; panel (**A**) represents change following tick parasitism in the treatment group, and panel (**B**) shows change following mock parasitism in the control group. Taxa are listed by genus, where possible, with family or next highest taxonomic group in parenthesis. * *q* < 0.05, ** *q* < 0.005, *** *q* < 0.0005.

For the lab experiment, the median number of reads per sample was 87,377 with a range of 63,716 to 101,481. The number of ASVs detected was 2807, and the number of taxa identified was 574. There was no effect of experimental grouping (i.e., control versus treatment; *F* = 0.005, *p* = 0.94), time (i.e., before versus after tick application, *F* = 0.06, *p* = 0.81), or tick parasitism (i.e., group-by-time interaction, *F* = 1.321, *p* = 0.27) on microbial taxonomic richness. There was, however, a significant effect of tick parasitism on fecal microbial community composition (i.e., a group-by-timepoint interaction; *F* = 3.35, *p* = 0.013), but there were no significant effects of either treatment group (*F* = 1.18, *p* = 0.25) or time (*F* = 0.90, *p* = 0.41) in isolation. As with the field data, there were significant effects of tick parasitism on the relative abundance of particular taxa. Tick-exposed mice showed significant overrepresentation of Muribaculaceae (*q* < 0.001), *Lactococcus* (*q* = 0.01), and *Arthromitus* (*q* = 0.02) relative to the pre-exposure timepoint, and significant underrepresentation of *Tuzzerella* (*q* = 0.03) (Figure 5A).

There were also significant changes in control mice before and after experimental infestation where *Helicobacter* (*q* = 0.03) was overrepresented in the second timepoint. and *Tuzzerella* (*q* = 0.02), Gastranaerophilales (*q* = 0.02), candidatus *Saccharimonas* (*q* = 0.03), NK4A214 group Oscillospiraceae (*q* = 0.002) were underrepresented in the second timepoint relative to the first (Figure 5B). We note that we recovered between seven and eighteen ticks per mouse per infestation (mean 12.8), but we note that it is likely that a number of ticks were undetected during daily cage cleaning; in a subsequent experiment (unpublished data), we found up to 71% more ticks during a second round of tick searching in each day’s cage remnants.

## 4. Discussion

We detected, in both a field study and laboratory experiment, that tick parasitism on rodents is associated with significant variation in the fecal microbiome. More specifically, experimental exposure to ticks resulted in a change in microbiome community composition in ways that were not seen in control mice, and with some changes paralleling differences between tick-parasitized and non-parasitized rodents in the field. The rodent gut microbiome is impacted by a wide variety of factors, and whereas certain lineages may be common to all members of a particular host species, heterogeneity among individuals and over time may mask other drivers of gut microbiome variation [43]. In our field study, the fecal microbiome varied by host species and sampling site, as well as tick parasitism, with substantial residual variation in microbiome composition unexplained by these variables; we did not test for variation by sex or age, and note that we did not have time-series data on tick parasitism for most of the field-sampled individuals. Given that the rodent species in our study have different ecological [44] and dietary characteristics [45,46], it is not surprising that their gut microbiota are different; prior studies have demonstrated that variation in habitat use, diet, and resource partitioning can impact the gut microbial communities in rodents [47,48,49], although variation among individuals may mask effects of diet and physiological state [43]. It is notable, however, that the same microbial taxon (Muribaculaceae) was associated with tick parasitism in both the field and lab studies, possibly suggesting at a mechanistic link between these two phenomena.

Recent research has highlighted links between the skin and gut microbiome, largely in reference to gut dysbiosis and effects on chronic skin conditions like psoriasis, atopic dermatitis and acne [22,50,51]. In humans, links between gut microbiome dysbiosis and skin pathologies have been described [52], and a weakened skin barrier to antigens can lead to immune-globulin E (IgE)-mediated food allergic response in the gut [53]. In another study, increased ultraviolet light exposure to the skin altered the fecal microbiome in ways that were consistent with oral vitamin D supplementation [54], further highlighting potential mechanisms by which impacts to the skin could manifest in changes to the gut microbiome. Mechanisms by which tick parasitism would elicit changes in the rodent fecal microbiome are unknown, and are beyond the scope of this study, but could involve immune response to tick salivary proteins or other impacts of tick feeding. Tick saliva is replete with proteins, lipids, and smaller peptides that are involved in mitigating pain/itch responses, promoting hemostasis, which can suppress the host’s immune response [55,56], and some of these host responses could have downstream impacts on the gut microbial community. For example, exposure to tick saliva, among other changes, promotes T helper 2 (Th2) cell response [57,58] and release of Th2-related cytokines [59], including interleukins 4 and 10, that are related with specific changes in the rodent gut microbiome [60].

One microbial taxon that was consistently represented by higher relative abundance in both the field and lab studies and across all three rodent taxa was the family Muribaculaceae (previously known as family S24-7) within the order Bacteroidales. Members of this family are common constituents of the rodent gut microbiome, representing as many as 83% of sequence reads assigned to the phylum Bacteriodota [61]. This family is characterized by metabolism of polysaccharides, including otherwise indigestible dietary fiber and mucin secreted by goblet cells in the gut [62,63]. Tick parasitism has been shown to elicit secretion of mucus from epithelial cells in the gut [64], which could result in increased relative abundance of microbial taxa that metabolize glycoproteins and other components of mucus. Related to disease and pathological processes, the high abundance of Muribaculaceae may be associated with better health outcomes because short-chain fatty acids, such as those produced by members of the Muribaculaceae, can have anti-inflammatory effects and lead to increased longevity in rodents [65]. In another study, two gut microbes, *Akkermansia miciniphilia* and a member of the Muribaculaceae (then known as family Bacteriodes S24-7), were associated with increased immunological response in mice [66]. With respect to parasitism, Kreisinger et al. [67] demonstrated that presence of tapeworms in the intestines of wild *Apodemus flavicollis* mice was associated with increased abundance of bacteria in the family S24-7 (now Muribaculaceae). We note that there were multiple sequence variants identified as members of the Muribaculaceae among our samples and studies, and we cannot be certain that all perform the same metabolic functions. Identification of any potential mechanisms by which tick parasitism would impact immune response in ways that could affect the gut microbiome, and specific taxa that may be affected by these immune responses, is worthy of future investigation.

With many hundreds of microbial taxa detected in a single fecal sample, it is prudent to interpret results of microbial community analyses with caution, particularly given the relatively small samples sizes in our field study, in particular where we were not able to control for effects of host sex, age, prior infection/parasitism status, genetics, or other aspects of individual-level variation. The amount of variation in wild rodent microbiota attributable to tick parasitism that we detected was far less than the variation between rodent species, and less than the variation attributable to sampling site, and we further cannot discount that potentially spurious or idiosyncratic changes in the microbiome were statistically attributed to tick parasitism. For example, Baxter et al. [43] reported that *Peromyscus* fecal microbiota varied substantially over time with samples taken from the same individual more than three days apart, showing more similarity to other individuals than to the initial sample. This result suggests that, at least for *P. leucopus*, gut microbiota change or turn over rapidly, and there is some risk of type I error when assessing the impacts of tick parasitism on the rodent gut microbiome, particularly in the field study. It is also possible that variation in the host microbiome affects parasitism by ticks, and some of the gut microbiome variation we describe in wild individuals with and without ticks may result from differences in likelihood of encountering or being parasitized by ticks. For example, elements of the skin microbiome can affect the attractiveness of certain host individuals to blood-feeding parasites [68], and infection with a microorganism may also influence blood-feeding by parasites, at least under some circumstances [69]. Similarly, the gut microbiome is linked to skin physiological function [21,22,23], and thus gut microbiome-mediated effects on skin could then impact parasitism by ticks. In cattle, differences in susceptibility to tick parasitism may be driven by variable immune system function in skin tissues [70]. Alternatively, gut microbiota can affect behavior [71], which can then lead to variation in likelihood of parasitism [25,72]. It is also possible that the composition of the internal microbiome could influence parasitism in other ways; Knutie [73] linked changes in the gut microbiome of eastern bluebirds following food supplementation to variation in parasitism by nest flies. Future studies where immune profiles are measured and quantified in response to tick parasitism and relative to changes in gut microbiome would help untangle the mechanistic relationship between tick parasitism and changes in the fecal microbiome.

In the lab experiment, we found that particular taxa in the microbiota of unparasitized mice changed significantly in relative abundance within a matter of several weeks, which is consistent with the findings of Baxter et al. [43]. We note that the characterization of tick parasitism for individuals in the field is based on a single point estimate, although tick parasitism status did not tend to change; for the subset of animals that were recaptured and checked for ticks on subsequent captures, there were two instances of change in tick parasitism status (both from parasitized to unparasitized, presumably because we removed the ticks on the previous occasion), and eight instances of the status remaining the same in subsequent captures (seven recaptures continued to be tick-free, whereas one individual was tick-parasitized on multiple occasions). This result is consistent with findings that (1) *P. leucopus* does not develop resistance to tick parasitism [74], and (2) that certain individuals in a population tend to have consistently higher tick burdens that others [74]. In other words, certain individuals may be more prone to tick parasitism than others [75,76]. It is also worth noting that interpretation of the field data, and comparisons to the lab experiment data, should account for the difference in the locus used to characterize microbiota. Use of different target loci within the 16S rRNA can affect the number of taxa identified in a sample [77,78], as can the taxonomic classification method [79]. Choice of sequencing platform (e.g., iSeq vs. MiSeq) can also affect detectable taxonomic resolution [80,81], but may be less impactful on overall microbiome characterization [82]. Whereas different sequence lengths between the field and lab samples preclude us from being able to directly assess similarity of taxa the species level, the iSeq is capable of characterizing taxa to genus and changes in abundance of particular taxa (e.g., Muribaculaceae) associated with tick parasitism should be fully comparable regardless of sequencing platform.

It is becoming increasingly apparent that the host and vector microbiome can influence transmission of vector-borne pathogens in a variety of ways, from facilitation or inhibition of pathogen acquisition to the probability of a vector feeding on a given host individual [16,20,21,22,23,24,25,83,84,85]. Our goal was to explore links between the host gut microbiome and tick parasitism by first describing variation and community composition of gut microbial communities in parasitized and unparasitized individuals of two wild rodent species, and second by tracking changes in the host microbiome following experimental exposure to ticks. Our results suggest that there may be particular gut microbial taxa that respond to physiological and/or immunological changes in hosts following tick parasitism, and that the overall gut microbial flora can be impacted by ectoparasite attachment and feeding; further studies that combine immunological assays with experimental tick parasitism and changes to the gut microbiome will elucidate such mechanisms. Whether these changes in gut microbial community composition impact host infection status to capacity to transmit vector-borne pathogens remains to be tested.

## 5. Conclusions

Tick parasitism on mammalian hosts, even in the absence of microbial pathogen transmission, results in an immune challenge that can affect physiological function [79]. Our data suggest that part of the host’s response to tick parasitism includes changes in the gut microbiome. Although the mechanisms by which the immunological response to ticks affect microbial community dynamics are unclear, it is reasonable to expect that immunologically mediated changes to epithelial cell function in the gut, possibly coordinated with responses by cells in the skin, would result in variation in resource availability for specific microbial taxa. We speculate that increased mucin production by goblet cells, stimulated by the host’s immune response to tick parasitism, facilitates reproduction of taxa in the Muribaculaceae and results in increased relative abundance of these lineages. We reach this conclusion based on the detection of consistent changes in abundance of this taxon in both observational and experimental studies of rodent fecal microbiota as part of broader changes in gut microbial community composition associated with parasitism by ticks.

## Figures and Tables

**Table 1:** Individual rodents sampled at each of four field sites in Virginia, USA, as well as tick parasitism information.

Sampling Location	Rodent Species	Individuals Sampled	Individuals with Ticks
Goochland	*Peromyscus leucopus*	24	9
Fort Barfoot	*Peromyscus leucopus*	14	3
	*Sigmodon hispidus*	7	3

## Data Availability

The original contributions presented in this study are included in the article. Further inquiries can be directed to the corresponding author.
